# Assessment of the BODE Index and Its Association With Inflammatory Mediators in Chronic Obstructive Pulmonary Disease (COPD) Patients

**DOI:** 10.7759/cureus.72172

**Published:** 2024-10-23

**Authors:** Kunjan Paresh Kumar Shah, Himani Prashanth Bhat, Mudra Kadam, Pransh Kachalia, Yesaswi Kuchi, Manik Siroha, Avanti Banerjee

**Affiliations:** 1 Department of General Medicine, Dr. N.D. Desai Faculty and Medical Research Institute, Nadiad, IND; 2 Department of General Medicine, Bala Gangadharanatha Swamy (BGS) Global Institute of Medical Sciences, Bengaluru, IND; 3 Department of General Medicine, Queen Alexandra Hospital, Portsmouth, GBR; 4 Department of General Medicine, Smt Mathurabai Bhausaheb Thorat (SMBT) Institute of Medical Sciences and Research Centre, Nashik, IND; 5 Department of General Medicine, Sri Venkateswara Institute of Medical Sciences-Sri Padmavathi Medical College for Women (SVIMS-SPMC(W), Tirupati, IND; 6 Department of General Medicine, Shree Guru Gobind Singh Tricentenary (SGT) University, Gurugram, IND; 7 Department of General Medicine, Queen Elizabeth Hospital Birmingham, Birmingham, GBR

**Keywords:** bode index, copd, c-reactive protein, gold criteria, inflammatory mediators, systemic inflammation

## Abstract

Background

Chronic obstructive pulmonary disease (COPD) is a respiratory condition impacting daily activities of susceptible individuals and increasing the risk of respiratory infections and cardiovascular disease. Body Mass Index, Airflow Obstruction, Dyspnea, and Exercise Capacity (BODE) index is applied clinically to measure the survival of COPD patients. Inflammatory mediators, including cytokines and chemokines, significantly contribute to the COPD pathology. In this study, the association between the BODE index and systemic inflammatory mediators in stable COPD patients was evaluated.

Methodology

This was a cross-sectional observational study performed on 85 clinically stable COPD patients and the GOLD criteria were used for the diagnosis. The demographics and clinical history of the patients were documented. The clinical assessment comprising the Modified Medical Research Council (MMRC) dyspnea scale, COPD Assessment Test (CAT) and BODE index was measured. The serum levels of systemic inflammatory mediators, tumor necrosis factor-alpha (TNF-α), C-reactive protein (CRP) and interleukin-6 (IL-6) were measured. The association between the BODE index and inflammatory markers was analyzed using Pearson’s correlation analysis.

Results

The majority of patients (61.2%) were in stage I BODE index and BODE index showed a significant correlation with GOLD stage severity (p=0.001). The CRP, TNF-α and IL-6 levels were increased in BODE stage IV when compared to stage III, II and I (p=0.001). The CRP (r=0.654; p=0.000), TNF-α (0.542; p=0.01) and IL-6 (r=0.498; p=0.02) showed significant correlation with BODE index.

Conclusion

The evaluation of the BODE index alongside systemic inflammatory markers is crucial for enhancing the management of COPD and subsequently improving patient outcomes.

## Introduction

Chronic obstructive pulmonary disease (COPD) is the most predominant respiratory disorder, which can be controlled with accurate therapeutic management. The clinical feature includes ongoing respiratory symptoms and obstructed airflow, which result from damage to airways and alveolar structures. These damages are primarily due to prolonged contact with noxious particles or gases [1]. Globally, the prevalence of COPD in 2020 was estimated to be 10.6%, with a staggering 480 million cases affecting both males and females [2]. The COPD prevalence in India is estimated to be 7.4% from the pooled sample estimate of 8,569 persons, and the rate is higher in males [3]. Tobacco smoking is widely recognized as the cardinal etiological factor for COPD in high- and medium-income countries. Meanwhile, exposure to biomass smoke continues to be a prevalent source of harmful substances, alpha-1 antitrypsin deficiency, asthma, and recurrent infections are also major contributors to the risk of COPD in low-income countries.

COPD is a complex and multifactorial condition that can profoundly affect an individual's quality of life and life expectancy. Chronic inflammation and damage to the airways and lungs can lead to various symptoms that worsen over time. Early identification and treatment of COPD are crucial to slow the disease's progression and improve patient outcomes [4]. People who have COPD often have a specific pattern of inflammation, with macrophages, neutrophils, T lymphocytes, and B lymphocytes are found in the secretions of the airways [5]. This inflammation pattern encompasses a network of both innate and acquired immune reactions, specifically cellular and humoral immunity, regulated by dendritic cell activation. Chronic inflammation orchestrates a cardinal role in the progression of COPD and has systemic effects. Tobacco smoke is primarily responsible for the high recruitment of inflammatory cells and their mediators in the lungs' tissues, which results in a constant inflammatory response. The increased amounts of neutrophils and T lymphocytes lead to tissue damage, along with an increase in the release of cytokines like interleukin-6 (IL-8), interleukin-8 (IL-8), and tumor necrosis factor-alpha (TNF-α) [6].

Currently, numerous techniques are employed to evaluate COPD severity and prognosis [7]. Body Mass Index, Airflow Obstruction, Dyspnea, and Exercise Capacity (BODE) index was introduced by Celli et al. and widely recognized as a valuable tool in assessing the severity of COPD and mortality prediction [8]. The BODE index takes into account several factors, including body mass index (BMI), airflow obstruction assessed by the Forced Expiratory Volume in One Second (FEV1), dyspnea scale estimated by the modified Medical Research Council (mMRC), and exercise capacity evaluated through the 6-minute walking distance (6MWD) examination [9]. In COPD individuals, the BODE index has been found to be a more reliable indicator of severity, acute exacerbation events, and mortality compared to the FEV1 [10]. In this backdrop, this study was conducted to evaluate the relationship between the BODE index and systemic inflammatory markers in patients with COPD.

## Materials and methods

This cross-sectional observational study was performed on 85 stable COPD patients attending the Department of General Medicine, Dr. N.D. Desai Faculty and Medical Research Institute, during the period between May 2023 and May 2024. This study was approved by the institutional ethical committee with protocol number NDDFMSR/IEC/176/2023.

Inclusion criteria

Both sexes over the age of 40, with clinically stable COPD and no signs of exacerbations over the last six weeks, were included. The study participants were identified according to GOLD criteria, specifically those with a post-bronchodilator FEV1/FVC (Forced vital capacity) ratio of less than 0.70, indicating the presence of persistent airflow limitation. The study also included patients who were exposed to biomass, second-hand smoke, or combined alcohol and tobacco use.

Exclusion criteria

Patients with COPD exacerbations and other respiratory pathologies such as asthma and lung emphysema were excluded from the study. Individuals with lung carcinoma, bronchiectasis, interstitial lung disease, and recent eye, thoracic, or abdominal surgery were also excluded from the study. Patients with inflammatory conditions like rheumatoid arthritis, malignancies, and other infectious conditions were also excluded from the study. This study also eliminated patients who were unable to do spirometry, unwilling to undergo echocardiography, and found to be free from any disease.

Study procedure

The demographic parameters and clinical features of the recruited COPD patients were documented. Dyspnea severity in COPD was assessed by the Modified Medical Research Council (MMRC) dyspnea scale and COPD Assessment Test (CAT) score. The patients underwent the 6-minute walking test (6MWT) and subsequent spirometry. BODE index is calculated using the patient's baseline body mass index (BMI), forced expiratory volume in the first second (FEV1), 6-minute walking distance, and MMRC dyspnea scale. FEV1, measured as a proportion of the expected value, is used in the GOLD staging algorithm. Each BODE stage was classified according to the BODE score [8], which varied between 0 and 10 points as shown in Table 1.

**Table 1:** BODE stages

BODE stage	BODE Score
1	0–2
2	3–4
3	5–6
4	7–10

A venous blood sample of 5 ml was taken and then centrifuged at a speed of 1,000 ×g for the duration of 15 minutes. The serum samples were carefully processed, and the aliquots were stored at -80°C for the measurement of inflammatory mediators. The C-reactive protein (CRP) was measured using a standard auto analyser and the tumor necrosis-alpha (TNF- α) and Inerleukin-6 (IL-6) levels were measured using a standard ELISA kit.

Data analysis

The data was analysed using SPSS statistical analysis software version 27 (IBM Corp., Armonk, NY, USA). The results were shown as mean, median and frequency. The comparison of variables between the BODE stages was done using One-way ANOVA. Pearson’s correlation analysis was done to study the association between the BODE index and serum inflammatory biomarkers. P<0.05 was measured as statistically significant.

## Results

Among 85 stable COPD patients, the serum inflammatory biomarkers were analyzed and correlated with the BODE index. The mean age of the COPD patients was 59.65±3.76 years, and the majority of the patients, 36.65%, were in the age group of 51-60 years. In this study, the majority of the COPD patients were males (72, 84.7%). The mean 6-minute walking distance was 345.76±109.12 meters, MMRC dyspnea grade was 2.10±0.76 and the mean BODE index score was 4.12±1.93. The majority of the patients (52, 61.2%) were in the stage I BODE index, followed by stage II (16, 18.8%), and stage III (12, 14.1%), respectively. The demographics and clinical features of the patients are presented in Table 2.

**Table 2:** Baseline characteristics of the COPD patients The data is shown as mean (SD) and frequency (%). COPD: Chronic obstructive pulmonary disease

Parameters	Values
Age in Years (mean±SD)	59.65±3.76
Age category (n, %)	
40-50	11 (12.94)
51-60	32 (36.65)
61-70	24 (24.24)
>70	18 (21.18)
Male / Female (n, %)	72 (84.7)/13 (15.3)
Pack years (mean±SD)	39.48±9.27
Duration of COPD in years (mean±SD)	5.12±1.32
BMI, Kg/m^2^ (mean±SD)	21.43±2.76
Forced Expiratory Volume 1 (FEV1) in Liters (mean±SD)	1.31±0.17
FEV1% predicted (mean±SD)	54.87±14.76
6-Minute Walking Distance in meters (mean±SD)	345.76±109.12
Modified Medical Research Council (MMRC) Dyspnea grade (mean±SD)	2.10±0.76
BODE Index	4.12±1.93
BODE Index stages (n, %)	
Stage I	52 (61.2)
Stage II	16 (18.8)
Stage III	12 (14.1)
Stage IV	5 (5.9)
C-reactive protein (CRP), mg/L (mean±SD)	7.08±1.24
Tumor necrosis Factor alpha (TNF)-α, pg/ml (mean±SD)	4.88±1.82
Interleukin-6 (IL-6), pg/ml (mean±SD)	3.53±1.94

A statistically significant correlation was established between the BODE index and the severity of GOLD stage COPD (p<0.001). In the BODE index stage III (5-6), the majority of the patients (56.2%) were in GOLD severe category, and in the BODE index stage IV (7-10), the majority of the patients (66.7%) were in GOLD very severe category. The results are shown in Table 3.

**Table 3:** Association between BODE index and GOLD severity in COPD patients Data is shown as frequency (%). * denotes significant, p<0.05, Chi-square test. GOLD 1: Mild, with an FEV1 ≥ 80% predicted; GOLD 2: Moderate, with an FEV1 50% ≤ FEV1 < 80% predicted; GOLD 3: Severe, with an FEV1 30% ≤ FEV1 < 50% predicted; and GOLD 4: Very severe, with an FEV1 < 30% predicted.

BODE Index Stages	GOLD Stage Severity				Chi-square P-value
	I (mild)	II (moderate)	III (severe)	IV (very severe)	
Stage I (0-2) (n,%)	20 (100%)	32 (74.4%)	0	0	X^2^=32.65; p=0.001*
Stage II (3-4) (n,%)	0	10 (23.3%)	6 (37.5%)	0	
Stage III (5-6) (n,%)	0	1 (2.3%)	9 (56.2%)	2 (33.3%)	
Stage IV (7-10) (n,%)	0	0	1 (6.3%)	4 (66.7%)	
Total	20 (100%)	43 (100%)	16 (100%)	6 (100%)	

In this study, the CRP level was higher in BODE stage IV (11.34±4.87 mg/L) when compared to stage III (8.45±2.54 mg/L), stage II (5.22±1.87mg/L) and stage I (3.54±1.21) respectively and it was significant (p=0.001). Correspondingly, the TNF-α (p=0.001) and IL-6 (p=0.001) levels were significantly greater in BODE stage IV when compared to stages III, II and I respectively. The comparison of inflammatory markers levels in BODE stages is shown in Table 4.

**Table 4:** Comparison of inflammatory markers levels in COPD patients with different BODE stages Data is shown as mean ± standard deviation. * denotes significant, p<0.05 (One-Way ANOVA). Reference range: CRP <3 mg/L; TNF-α <2.8 pg/ml; IL-6 <1.8 pg/ml CRP: C-reactive protein; TNF-α: Tumor necrosis factor alpha; IL-6: Interleukin-6

Inflammatory markers (mean±SD)	BODE stage				F value	P value
	Stage I (n=52)	Stage II (n=16)	Stage III (n=12)	Stage IV (n=5)		
CRP (mg/L)	3.54±1.21	5.22±1.87	8.45±2.54	11.34±4.87	125.65	0.001*
TNF-α (pg/ml)	2.39±0.66	4.97±0.86	5.55±1.03	7.38±2.54	59.42	0.001*
IL-6 (pg/ml)	1.14±0.33	2.94±0.76	4.84±1.12	6.50±1.87	87.65	0.001*

In this study, there was a significant association among CRP (r=0.654; p=0.000), TNF-α (0.542; p=0.01) and IL-6 (r=0.498; p=0.02). Meanwhile, CRP showed a strong correlation with the BODE index when compared to TNF-α and Il-6. The correlation between the BODE index and stages and inflammatory markers levels is shown in Table 5.

**Table 5:** Correlation of inflammatory markers with BODE index in COPD patients * denotes significant, p<0.05 (Pearson’s correlation) CRP: C-reactive protein; TNF-α: Tumor necrosis factor alpha; IL-6: Interleukin-6

Inflammatory markers	Pearson’s correlation coefficient (r)	P-value
CRP (mg/L)	0.654	0.000*
TNF-α (pg/ml)	0.542	0.01*
IL-6 (pg/ml)	0.498	0.02*

## Discussion

The BODE index is a multi-dimensional grading system that considers the following four important aspects of COPD assessment: exercise ability, dyspnea, airflow obstruction, and BMI. It is a crucial tool for forecasting COPD patients' deaths and disease progression. When compared to other established metrics like FEV1 alone, the BODE index offers a more thorough assessment of the severity of COPD. The BODE index enables a more individualized approach to COPD management and treatment by taking into account several variables [11].

In addition to the BODE index, another important aspect to consider in evaluating COPD is the involvement of inflammatory mediators in disease progression. These mediators mediate a crucial function in the chronic inflammation and tissue damage seen in COPD individuals [12]. Important inflammatory mediators in COPD include IL-6, IL-8, and TNF-α. Numerous lung cells, such as neutrophils, epithelial cells, and macrophages, release these mediators in reaction to exposure to cigarette smoke and other environmental stimuli. TNF-α, for example, is known to promote inflammation and tissue destruction in the lungs, while IL-6 and IL-8 play mediate immune cell recruitment to the inflammation site [13]. Further, there is an elevated level of CRP during COPD exacerbations, which leads to symptoms worsening [14,15]. Thus, our study examined the association between the BODE index and systemic inflammatory mediators in stable COPD patients.

The average age of the patients in this study was 59.65±3.76, with a peak incidence of the disease in the 5th-6th decade. Similarly, in a study done by Pandey et al., the mean age of COPD patients was 57.41±9.88 years [16]. The incidence of COPD is significantly higher in individuals over the age of 60, with rates being 2-3 times greater compared to younger age groups. The greater occurrence of COPD mentioned in the older population can be ascribed to age-related variations in lung structure and function, which increase the susceptibility to developing COPD [17].

The present study found a male predominance among COPD patients, comprising 84.7% of the study participants than females with 15.3%. Likewise, Meshram et al.’s study described that the frequency of COPD was greater in males than females (86% vs. 14%). The perception of COPD as being more prevalent in males may have led to a lack of diagnosis in females, resulting in an underestimation of the impact of the disease, as well as the higher incidence of smoking habits in males. The study reported a mean BODE index of 4.12±1.93 and found that the stage I BODE index was most common in 61.2% of COPD patients [18]. This finding aligns with a study performed by Khan et al. - the majority of the COPD patients, 66.5%, were in the BODE index quartile 1 [19]. In another study done by Liu et al., 40.8% of COPD patients were in the BODE index quartile 1 [20].

The BODE index and GOLD staging systems demonstrate comparable clinical efficacy in forecasting the risk of exacerbation among the ambulatory COPD patients. Both the indices demonstrated similar effects on the risk of exacerbation during the follow-up period of one year [21]. Variables not included in either system were identified as the important predictors of exacerbation risk. In our study, there was a marked correlation between the BODE index and the severity of COPD as classified by the GOLD stage (p<0.001). Increased severity of airflow obstruction leads to the corresponding increase in the BODE index score. This can be attributed to the inclusion of FEV1 in both the GOLD grade and the BODE index. In Pandey et al.’s study, there was an increased BODE score in the GOLD stage IV (very severe) and it was found to be significant (p<0.001) [16]. So this implicates a significant correlation among the GOLD stage and the BODE index and both can be used in combination to predict the severity and prognosis in COPD patients.

TNF-α mediates a cardinal role in the control of multiple immune responses and serves as a potent proinflammatory cytokine. During a COPD exacerbation, excessive production of TNF occurs, which plays a significant role in attracting and activating immune cells, specifically neutrophils and macrophages. Additionally, it stimulates the secretions of other inflammatory cytokines, further intensifying the inflammatory response and causing damage to the tissues, and restricting airflow [22]. In this study, the increased level of TNF-α was observed in BODE index stage IV when compared to stages III, II and I, respectively (7.38±2.54 pg/ml vs. 5.55±1.03, 4.97±0.86 and 2.39±0.66 pg/ml; p=0.001). Similar to our report, Khan et al.’s study observed increased TNF-α levels in BODE index quartile III of high severity when compared to quartiles II and I (4.72±2.07 pg/ml vs. 4.21±1.87 pg/ml and 3.94±2.02 pg/ml) [19]. IL-6 plays a vital role in the production of acute-phase proteins when it interacts with liver cells. Mounting studies have found that IL-6 levels in the peripheral blood of COPD patients were noticeably greater, and this increase was linked to FEV1 [23]. Meanwhile, in the present study, IL-6 levels also showed a similar pattern with increased levels of IL-6 in the BODE index stage IV when compared to stages III, II and I (6.50±1.87 pg/ml vs. 4.84±1.12, 2.94±0.76 and 1.14±0.33 pg/ml; p=0.001). Likewise, in a research done by Khan et al., IL-6 level was increased in BODE index quartile III when compared to the quartiles II and I (2.88±1.82 pg/ml vs. 1.97±1.04 pg/ml and 1.21±0.83 pg/ml; p<0.001) [19]. CRP is one of the important blood markers in COPD patients for the evaluation of the degree of severity. In patients with COPD, elevated CRP levels have been associated with reduced lung function, diminished physical activity, and a lower quality of life. Additionally, elevated CRP levels serve as a strong indicator of all-cause mortality [24]. In addition to COPD itself, smoking, which is the generally perceived risk factor for the disease, also contributes to an increase in serum CRP levels. In our study, the increased level of CRP was observed in BODE index stage IV when compared to stages III, II and I respectively (11.34±4.87 mg/L vs. 8.45±2.54, 5.22±1.87 and 3.54±1.21 mg/L; p=0.001). Likewise, in a research done by Ghobadi et al., CRP level was increased in BODE index quartile IV when compared to the quartiles III, II and I (10.88±10.53 mg/L vs. 7.91±7.15, 3.87±5.88, 3.50±4.87 mg/L; p=0.03) [25].

In this investigation, a significant association was observed among CRP (r=0.654; p=0.000), TNF-α (0.542; p=0.01), and IL-6 (r=0.498; p=0.02). In a research documented by Pandey et al., a significant correlation was observed between the BODE index and CRP, with a correlation coefficient of r=0.504 and a p-value of less than 0.0001 [16]. In addition, Yazici et al. [26] reported a strong association between TNF-α (r=0.367; p=0.004) and IL-6 (r=0.541; p=0.001) and BODE index and a recent study conducted by Hemalatha et al. [27] also reported a strong association between the BODE index and CRP (r=0.359; p=0.002).

Limitations

Potential limitations of the study include a small sample size and research being conducted at a single center. Our investigation has focused on a restricted set of inflammatory markers, and it is necessary to assess those using new variables.

## Conclusions

In summary, the present investigation conclusively establishes a substantial association between the BODE index and inflammatory mediators in individuals with COPD. Hence, the panel of systemic inflammatory markers is recommended in standard clinical practice to assess the severity of COPD. The investigation of the interaction between the BODE index and inflammatory mediators may also facilitate the advancement of specific treatments that target the fundamental inflammation in COPD. Undoubtedly, ongoing study in this field has the capacity to completely transform the treatment of COPD and enhance results for patients.
